# The Truncated Cauchy Power Family of Distributions with Inference and Applications

**DOI:** 10.3390/e22030346

**Published:** 2020-03-17

**Authors:** Maha A. Aldahlan, Farrukh Jamal, Christophe Chesneau, Mohammed Elgarhy, Ibrahim Elbatal

**Affiliations:** 1 Department of Statistics, College of Science, University of Jeddah, Jeddah 23218, Saudi Arabia; maal-dahlan@uj.edu.sa; 2 Department of Statistics, Government Postgraduate College Der Nawab Bahawalpur, Punjab 63351, Pakistan; drfarrukh1982@gmail.com; 3 Department of Mathematics, LMNO, Campus II, Science 3, Université de Caen, 14032 Caen, France; 4 Valley High Institute for Management Finance and Information Systems, Obour, Qaliubia 11828, Egypt; m_elgarhy85@sva.edu.eg; 5 Department of Mathematics and Statistics-College of Science, Imam Muhammad ibn Saud Islamic University, Riyadh 11432, Saudi Arabia; 6 Department of Mathematical Statistics, Faculty of Graduate Studies for Statistical Research, Cairo University, Benha 13513, Egypt; iielbatal@imamu.edu.sa

**Keywords:** Cauchy distribution, truncated distribution, general family of distributions, entropy, estimation, simple random sampling, ranked set sampling, data analysis, 60E05, 62E15, 62F10

## Abstract

As a matter of fact, the statistical literature lacks of general family of distributions based on the truncated Cauchy distribution. In this paper, such a family is proposed, called the truncated Cauchy power-G family. It stands out for the originality of the involved functions, its overall simplicity and its desirable properties for modelling purposes. In particular, (i) only one parameter is added to the baseline distribution avoiding the over-parametrization phenomenon, (ii) the related probability functions (cumulative distribution, probability density, hazard rate, and quantile functions) have tractable expressions, and (iii) thanks to the combined action of the arctangent and power functions, the flexible properties of the baseline distribution (symmetry, skewness, kurtosis, etc.) can be really enhanced. These aspects are discussed in detail, with the support of comprehensive numerical and graphical results. Furthermore, important mathematical features of the new family are derived, such as the moments, skewness and kurtosis, two kinds of entropy and order statistics. For the applied side, new models can be created in view of fitting data sets with simple or complex structure. This last point is illustrated by the consideration of the Weibull distribution as baseline, the maximum likelihood method of estimation and two practical data sets wit different skewness properties. The obtained results show that the truncated Cauchy power-G family is very competitive in comparison to other well implanted general families.

## 1. Introduction

The general version of the truncated Cauchy distribution is defined by the following cumulative distribution function (cdf):

$$F_{\left(a,b\right)}\left(x;\mu ,\theta \right)=\frac{\arctan[(x-\mu )/\theta ]-\arctan[(a-\mu )/\theta ]}{\arctan[(b-\mu )/\theta ]-\arctan[(a-\mu )/\theta ]},\;x\in \left(a,b\right),$$

where (a,b)∈R∪{−∞,+∞}, μ∈R and θ>0 (including the so-called half-Cauchy distribution defined with a=0 and b=+∞). It was introduced by [1], with a discussion on the symmetric standard case characterized by the following configuration: a=−b, μ=0 and θ=1. In comparison to the well-known Cauchy distribution, it has finite moments when *a* and *b* are finite, and it offers a more realistic alternative for modelling purposes since most of the practical data sets are defined on a finite range of values, which can often be determined based on historical records. The main mathematical properties of the truncated Cauchy distribution can be found in [2,3,4].The statistical features of the related model can be found in [1,3,5], with applications as well (stock returns, exchange rate data…). Also, the computational aspects of the truncated Cauchy distribution via the R software are addressed in [6,7].

By the use of well-known general families of distributions, one can extend the truncated Cauchy distribution in multiple theoretical or applied directions. For instance, one can use the exp-G family proposed by [8], the Kumaraswamy-G family introduced by [9], the beta-G family developed by [10], the Marshall-Olkin-G family proposed by [11], the Weibull-G family developed by [12,13], the transmuted-G family developed by [14], the gamma-G family proposed by [15], the inverse exponential-G family proposed by [16], the sine-G family introduced by [17], and the truncated inverted Kumaraswamy-G family proposed by [18]. The idea behind this general families is to transform or add (one or several) parameters to a baseline distribution in order to improve its global flexibility, with the aim to gain on the fitting of the resulting models. In the special case of the half-Cauchy distribution, such extensions have been explored by [19] via the the Marshall-Olkin-G family, by [20] via the beta-G family, by [21] via the Kumaraswamy-G family and by [22] via the Weibull-G family and by [23] via the gamma-G family. However, to the best of our knowledge, the extensions of the truncated Cauchy distribution with finite *a* and *b* can be performed in a similar manner (but remains to study in an extensive way).

Another way to exploit the features of the truncated Cauchy distribution is to use it as a generator of new families of distributions. In the special case of the half-Cauchy distribution, this is performed by [24] which introduced the generalized odd half-Cauchy-G (GOHC-G) family defined by the following cdf:

$$F\left(x;\alpha ,\beta \right)=F_{\left(0,+\infty \right)}\left[\frac{G{\left(x;\xi \right)}^{\alpha }}{1-G{\left(x;\xi \right)}^{\alpha }}\right]=\frac{2}{\pi }\arctan\left[\frac{G{\left(x;\xi \right)}^{\alpha }}{1-G{\left(x;\xi \right)}^{\alpha }}\right],$$

where α>0 and G(x;ξ) denotes the cdf of a univariate continuous distributions with parameter vector denoted by ξ. A twin family is given by the odd power Cauchy-G (OPC-G) introduced by [25] and defined by the following cdf:

$$F_{*}\left(x;\alpha ,\beta \right)=F_{\left(0,+\infty \right)}\left[{\left(\frac{G(x;\xi )}{1-G(x;\xi )}\right)}^{\alpha }\right]=\frac{2}{\pi }\arctan\left[{\left(\frac{G(x;\xi )}{1-G(x;\xi )}\right)}^{\alpha }\right].$$

These two families show practical merits, producing skewness for symmetrical distributions, constructing heavy-tailed distributions, generating distributions with various shapes on their probability functions, providing better fits than other families of distributions under the same baseline…. However, the study of their theoretical properties is not an easy task. One common drawback remains in the complexity of the corresponding probability functions, which can afraid the occasional practitioner, and the mathematical complexity of some related measures. In particular, the corresponding probability density function has a linear decomposition with non-closed form coefficients with sophisticated recurrence structures (mainly based on technical results in [26]). Thus, to the best of our knowledge, the statistical literature lacks on simple general family of distributions involving the arctangent function.

In this paper, we offer a comprehensible alternative by introducing the truncated Cauchy power-G (TCP-G) family. It is defined on the basis on the truncated Cauchy distribution on the interval (0,1) and the exp-G family. Indeed, the cdf of the TCP-G family is given by

(1) $$\begin{array}{c} F\left(x;\alpha ,\xi \right)=F_{\left(0,1\right)}\left[G{\left(x;\xi \right)}^{\alpha }\right]=\frac{4}{\pi }\arctan\left[G{\left(x;\xi \right)}^{\alpha }\right],\;x\in R, \end{array}$$

where α>0 and, again, G(x;ξ) denotes the cdf of a univariate continuous distributions with parameter vector denoted by ξ. As immediate remark, the cdf of the TCP-G family has a simple expression, with an immediate series expansion, which is not the case for the GOHC-G or OPC-G families. The related probability functions can be deduced easily, with tractable expressions and immediate series expansions. Thus, the main properties of the TCP-G family can be derived, including the analyzes of the shapes of the probability and hazard rate functions, as well as their asymptotic properties, the quantile function, moments and related functions, several measures of skewness and kurtosis, Rényi and *q*-entropies and order statistics. Then, the estimation of the TCP-G model parameters is investigated by the maximum likelihood method, with an emphasis on the one defined with the Weibull distribution as baseline. To evaluate the performance of the obtained estimates, two sampling schemes are considered, namely the simple random sampling and the ranked set sampling. As expected, nice numerical results are obtained for both. Then, two practical data sets are employed to show the modelling ability of the TCP-G family. More precisely, with the consideration of the Weibull distribution as baseline, we show that the TCP-G family generates very competitive models compared with other widely known general families, such as the Kumaraswamy-G and beta-G families with however one more parameter.

The rest of the paper is organized as follows. In Section 2, more mathematical backgrounds are given on the TCP-G family. Its most notable properties are presented in Section 3. The estimation of the model parameters is discussed in Section 4. Section 5 is devoted to the applied part. Some concluding remarks and perspectives are communicated in Section 6.

## 2. The TCP-G Family

This section is devoted to the description of the main probability functions of the TCP-G family, namely the probability density, hazard rate and quantile functions, with discussions on some of their analytical properties. A special member of the family is presented as example.

### 2.1. Probability Density Function

The probability density function (pdf) of the TCP-G family can be obtained upon differentiation the cdf given by (1). Thus, it is obtained as

(2) $$\begin{array}{c} f\left(x;\alpha ,\xi \right)=\frac{4\alpha }{\pi }\frac{g\left(x;\xi \right)G{\left(x;\xi \right)}^{\alpha -1}}{1+G{\left(x;\xi \right)}^{2\alpha }},\;x\in R, \end{array}$$

where g(x;ξ) denotes the corresponding pdf to G(x;ξ).

Some analytical properties of f(x;α,ξ) are as follows.

When G(x;ξ)→0, we get $f\left(x;\alpha ,\xi \right)∼\left(4\alpha /\pi \right)g\left(x;\xi \right)G{\left(x;\xi \right)}^{\alpha -1}$. We thus observe an effect of the parameter α on the asymptotic properties of f(x;α,ξ). For instance, by assuming that g(x;ξ) is bounded, if α>1, we have f(x;α,ξ)→0 and if α∈(0,1), we have f(x;α,ξ)→+∞. Also, when G(x;ξ)→1, we get f(x;α,ξ)∼(2α/π)g(x;ξ).

The critical point(s) of f(x;α,ξ) is (are) of interest for the uni/multimodality analysis and, a fortiori, modelling perspectives. Thus, a critical point $x_{c}$ of f(x;α,ξ) satisfies the non-linear equation given by ${\left\{\log\left[f\left(x;\alpha ,\xi \right)\right]\right\}}^{\prime }{|}_{x=x_{c}}=0$, i.e.,

$$\begin{array}{c} \frac{g^{\prime }\left(x_{c};\xi \right)}{g(x_{c};\xi )}+\left(\alpha -1\right)\frac{g(x_{c};\xi )}{G(x_{c};\xi )}-2\alpha \frac{g\left(x_{c};\xi \right)G{\left(x_{c};\xi \right)}^{2\alpha -1}}{1+G{\left(x_{c};\xi \right)}^{2\alpha }}=0. \end{array}$$

The nature of $x_{c}$ depends on the position of the value of $\eta ={\left\{\log\left[f\left(x;\alpha ,\xi \right)\right]\right\}}^{\prime \prime }{|}_{x=x_{c}}$ about 0, i.e.,

$$\begin{array}{cc} \eta & =\frac{g^{\prime \prime }\left(x_{c};\xi \right)g\left(x_{c};\xi \right)-g^{\prime }{\left(x_{c};\xi \right)}^{2}}{g{\left(x_{c};\xi \right)}^{2}}+\left(\alpha -1\right)\frac{g^{\prime }\left(x_{c};\xi \right)G\left(x_{c};\xi \right)-g{\left(x_{c};\xi \right)}^{2}}{G{\left(x_{c};\xi \right)}^{2}}\\ \; & +8\alpha^{3}\frac{g^{\prime }{\left(x_{c};\xi \right)}^{2}G{\left(x_{c};\xi \right)}^{4\alpha -2}}{{\left[1+G{\left(x_{c};\xi \right)}^{2\alpha }\right]}^{2}}-4\alpha^{2}\frac{g^{\prime \prime }\left(x_{c};\xi \right)G{\left(x_{c};\xi \right)}^{2\alpha -1}}{1+G{\left(x_{c};\xi \right)}^{2\alpha }}-4\alpha^{2}\left(2\alpha -1\right)\frac{g^{\prime }{\left(x_{c};\xi \right)}^{2}G{\left(x_{c};\xi \right)}^{2\alpha -2}}{1+G{\left(x_{c};\xi \right)}^{2\alpha }}. \end{array}$$

Hence, if η>0, then $x_{c}$ is a local minimum, if η<0 then $x_{c}$ is a local maximum and if η=0, then $x_{c}$ is an inflexion point. There is no closed-form for $x_{c}$ or η; mathematical softwares are required to provide numerical evaluations for $x_{c}$ or η.

### 2.2. Hazard Rate Function

The hazard rate function (hrf) of the TCP-G family is defined by h(x;α,ξ)=f(x;α,ξ)/[1−F(x;α,ξ)], i.e.,

(3) $$\begin{array}{c} h\left(x;\alpha ,\xi \right)=\frac{4\alpha }{\pi }\frac{g\left(x;\xi \right)G{\left(x;\xi \right)}^{\alpha -1}}{\left[1+G{\left(x;\xi \right)}^{2\alpha }\right]\left\{1-\left(4/\pi \right)\arctan[G{\left(x;\xi \right)}^{\alpha }]\right\}},\;x\in R. \end{array}$$

We present some of its immediate analytical properties below.

When G(x;ξ)→0, we get $h\left(x;\alpha ,\xi \right)∼f\left(x;\alpha ,\xi \right)∼\left(4\alpha /\pi \right)g\left(x;\xi \right)G{\left(x;\xi \right)}^{\alpha -1}$. Hence, as for f(x;α,ξ), the parameter α plays an important role on the asymptotic properties of h(x;α,ξ). When G(x;ξ)→1, by using the following equivalence: when y→1, arctan(y)∼π/4−(1−y)/2, we get $h\left(x;\alpha ,\xi \right)∼\alpha g\left(x;\xi \right){\left[1-G\left(x;\xi \right)\right]}^{-1}$.

The possible shapes for h(x;α,ξ) are of interest from the modelling point of view. Here, we only discuss the critical point(s) of this function. Thus, a critical point $x_{o}$ of h(x;α,ξ) satisfies the non-linear equation given by ${\left\{\log\left[h\left(x;\alpha ,\xi \right)\right]\right\}}^{\prime }{|}_{x=x_{o}}=0$, i.e.,

$$\begin{array}{cc} \; & \frac{g^{\prime }\left(x_{o};\xi \right)}{g(x_{o};\xi )}+\left(\alpha -1\right)\frac{g(x_{o};\xi )}{G(x_{o};\xi )}-2\alpha \frac{g\left(x_{o};\xi \right)G{\left(x_{o};\xi \right)}^{2\alpha -1}}{1+G{\left(x_{o};\xi \right)}^{2\alpha }}\\ \; & +\frac{4\alpha }{\pi }\frac{g\left(x_{o};\xi \right)G{\left(x_{o};\xi \right)}^{\alpha -1}}{\left[1+G{\left(x_{o};\xi \right)}^{2\alpha }\right]\left\{1-\left(4/\pi \right)\arctan[G{\left(x_{o};\xi \right)}^{\alpha }]\right\}}=0. \end{array}$$

The nature of $x_{o}$ depends on the position of the value of $\upsilon ={\left\{\log\left[h\left(x;\alpha ,\xi \right)\right]\right\}}^{\prime \prime }{|}_{x=x_{o}}$ about zero. We omit to express it for the sake of place. Again, there is no closed-form for $x_{o}$ or υ, but the use of a mathematical software can help to evaluate them.

### 2.3. Quantile Function

The quantile function (qf) of the TCP-G family is the functional solution Q(u;α,ξ) of the following non-linear equation: F(Q(u;α,ξ);α,ξ)=u for any u∈(0,1), i.e., $\left(4/\pi \right)\arctan[G{\left(Q\left(u;\alpha ,\xi \right);\xi \right)}^{\alpha }]=u$. After some algebra, we get

(4) $$\begin{array}{c} Q\left(u;\alpha ,\xi \right)=Q_{*}\left\{{\left[\tan\left(\frac{\pi }{4}u\right)\right]}^{1/\alpha };\xi \right\},\;u\in \left(0,1\right), \end{array}$$

where $Q_{*}\left(u;\xi \right)$ denotes the qf corresponding to G(x;ξ).

The standard quantiles can be deduced. Among them, the median defined by M(α,ξ)=Q(1/2;α,ξ) plays an important role.

The quantile function is useful to simulate values from distributions belonging to the TCP-G family. Indeed, for a given baseline cdf G(x;ξ), from *n* values $u_{1},\ldots ,u_{n}$ randomly and independently obtained from the uniform distribution over (0,1), then $x_{1},\ldots ,x_{n}$ with $x_{i}=Q\left(u_{i};\alpha ,\xi \right)$ are *n* values randomly and independently obtained from the corresponding TCP-G distribution.

Furthermore, the quantile function allows defining some skewness and kurtosis measures. They have the advantage to always exist contrary to those defined with moments.

If $Q_{*}\left(u;\xi \right)$ has not an analytical expression but can be expressed by a power expansion series (such as the qf of the normal distribution), one can determine a power expansion series for Q(u;α,ξ) by proceeding as in Section 3.4 of [25].

### 2.4. Example: The Truncated Cauchy Power Weibull Distribution

By construction, the TCP-G family is rich and contains numerous new distributions with a potential interest from a statistical point of view (with different supports, numbers of parameters, properties…). Here, we focus our attention on the member of the TCP-G family defined with the Weibull distribution as baseline. For the purpose of this paper, it is called the truncated Cauchy power Weibull (TCPW) distribution.

In this study, the cdf of the Weibull distribution is defined by $G\left(x;\lambda ,\theta \right)=1-e^{-\lambda x^{\theta }}$, x>0, where λ,θ>0, so ξ=(λ,θ), and the corresponding pdf is obtained as $g\left(x;\lambda ,\theta \right)=\lambda \theta x^{\theta -1}e^{-\lambda x^{\theta }}$, x>0. Hence, by substituting this cdf into (1), the TCPW distribution is defined by the following cdf:

(5) $$\begin{array}{c} F\left(x;\alpha ,\lambda ,\theta \right)=\frac{4}{\pi }\arctan\left[{\left(1-e^{-\lambda x^{\theta }}\right)}^{\alpha }\right],\;x>0. \end{array}$$

Thus defined, α and θ are two positive shape parameters and λ is a positive scale parameter. Also, the corresponding pdf is given by

(6) $$\begin{array}{c} f\left(x;\alpha ,\lambda ,\theta \right)=\frac{4\alpha \lambda \theta }{\pi }\frac{x^{\theta -1}e^{-\lambda x^{\theta }}{\left(1-e^{-\lambda x^{\theta }}\right)}^{\alpha -1}}{1+{\left(1-e^{-\lambda x^{\theta }}\right)}^{2\alpha }},\;x>0, \end{array}$$

**Figure 1:**
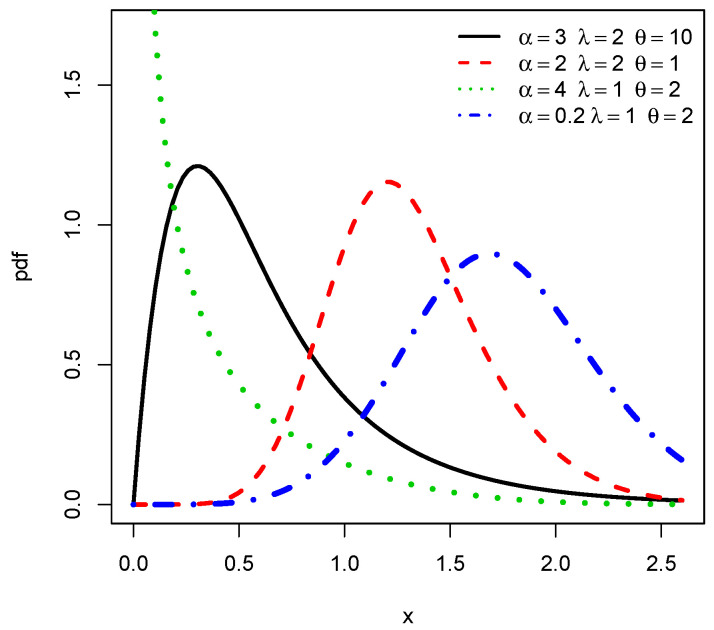
Plots of the pdf of the TCPW distribution for various values of the three parameters.

As immediate facts, the following asymptotic properties hold. When x→0, we get $f\left(x;\alpha ,\lambda ,\theta \right)∼\left(4\alpha \lambda^{\alpha }\theta /\pi \right)x^{\alpha \theta -1}$. Hence, if αθ<1, f(x;α,λ,θ) tends to +∞, if αθ=1, f(x;α,λ,θ) tends to $4\lambda^{\alpha }/\pi$, and if αθ>1, f(x;α,λ,θ) tends to 0. When x→+∞, we have $f\left(x;\alpha ,\lambda ,\theta \right)∼\left(2\alpha \lambda \theta /\pi \right)x^{\theta -1}e^{-\lambda x^{\theta }}$, which tends to 0 for all the values of the parameters. Numerical investigations of the critical points show that the TCPW distribution is mainly unimodal: only one maximum is reached. Figure 1 illustrates the possible shapes for f(x;α,λ,θ) by considering the following four sets of parameters as (α,λ,θ): (3,2,10), (2,2,1), (4,1,2) and (0.2,1,2). We see that f(x;α,λ,θ) can be left, right skewed, near symmetrical and reverse J shaped.

The hrf of the TCPW distribution is obtained as

(7) $$\begin{array}{c} h\left(x;\alpha ,\lambda ,\theta \right)=\frac{4\alpha \lambda \theta }{\pi }\frac{x^{\theta -1}{\left(1-e^{-\lambda x^{\theta }}\right)}^{\alpha -1}}{\left[1+{\left(1-e^{-\lambda x^{\theta }}\right)}^{2\alpha }\right]\left\{1-\left(4/\pi \right)\arctan\left[{\left(1-e^{-\lambda x^{\theta }}\right)}^{\alpha }\right]\right\}},\;x>0. \end{array}$$

The following asymptotic properties hold. When x→0, we get $h\left(x;\alpha ,\lambda ,\theta \right)∼\left(4\alpha \lambda^{\alpha }\theta /\pi \right)x^{\alpha \theta -1}$. Therefore, if αθ<1, h(x;α,λ,θ) tends to +∞, if αθ=1, h(x;α,λ,θ) tends to $4\lambda^{\alpha }/\pi$, and if αθ>1, h(x;α,λ,θ) tends to 0.

Also, when x→+∞, we have $h\left(x;\alpha ,\lambda ,\theta \right)∼\alpha \lambda \theta x^{\theta -1}$. Hence, if θ<1, h(x;α,λ,θ) tends to 0, if θ=1, h(x;α,λ,θ) tends to αλθ, and if θ>1, h(x;α,λ,θ) tends to +∞.

**Figure 2:**
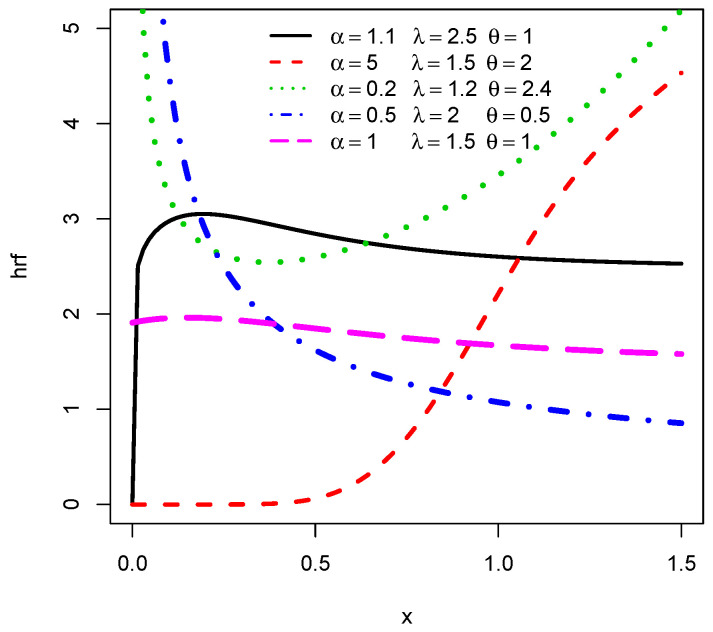
Plots of the hrf of the TCPW distribution for various values of the three parameters.

Numerical investigations of the critical points can be performed for h(x;α,ξ). For a visual approach, Figure 2 illustrates the possible shapes for h(x;α,λ,θ) by considering the following five sets of parameters as (α,λ,θ): (1.1,2.5,1), (5,1.5,2), (0.2,1.2,2.4), (0.5,2,0.5) and (1,1.5,1). We notice again that the TCPW distribution is a very flexible distribution, having all possible monotonic and non-monotonic hazard rate shapes, such as increasing, decreasing, decreasing-increasing-decreasing, constant, bathtub and upside-down bathtub shapes.

After some algebra, the quantile function of the TCPW distribution is defined by

(8) $$\begin{array}{c} Q\left(u;\alpha ,\lambda ,\theta \right)={\left[-\frac{1}{\lambda }\log\left(1-{\left[\tan\left(\frac{\pi }{4}u\right)\right]}^{1/\alpha }\right)\right]}^{1/\theta },\;u\in \left(0,1\right). \end{array}$$

This tractable expression is an undeniable plus to simulate values from the TCPW distribution and to defined skewness and kurtosis measures, wherever the existence or not of moments. These points will be discussed later.

## 3. Notable Properties

In this section, some notable properties of the TCP-G family, and of the TCPW distribution in particular, are derived.

### 3.1. Linear Representations

Simple expansion series for the pdf and cdf of the TCP-G family are obtained according to the cdf and pdf of the exponentiated-G family by [8] given by $G_{\gamma }\left(x;\xi \right)=G{\left(x;\xi \right)}^{\gamma }$ and $g_{\gamma }\left(x;\xi \right)=\gamma g\left(x;\xi \right)G{\left(x;\xi \right)}^{\gamma -1}$, where γ>0. The interest of such expansions series is mainly for practical purposes: the determination of some properties of the TCP-G family via such expansions can be more efficient than computing those directly by numerical integration involving the corresponding pdf (which is well-known to prone to rounding off errors).

Since $G{\left(x;\xi \right)}^{\alpha }\in \left(0,1\right)$, owing to the well-known series decomposition of the arctangent function, we have the following series expansion for F(x;α,ξ):

(9) $$\begin{array}{c} F\left(x;\alpha ,\xi \right)=\frac{4}{\pi }\sum_{k=0}^{+\infty }\frac{{\left(-1\right)}^{k}}{2k+1}G_{\alpha (2k+1)}\left(x;\xi \right). \end{array}$$

Upon differentiation of F(x;α,ξ), a series expansion for f(x;α,ξ) follows:

(10) $$\begin{array}{c} f\left(x;\alpha ,\xi \right)=\frac{4}{\pi }\sum_{k=0}^{+\infty }\frac{{\left(-1\right)}^{k}}{2k+1}g_{\alpha (2k+1)}\left(x;\xi \right). \end{array}$$

One can remark that the coefficients in these series expansions are readily computed numerically using any standard mathematical software. Also, in any numerical calculations using these series expansions, infinity should be substituted by a large integer number. In this sense, some properties of the exponentiated-G family can be useful to determine those of the TCP-G family, as developed for the moments and related functions in the next section.

In this study, we will use them to provide series expansions for the moments and related functions. Also, for a given baseline cdf G(x;ξ), we can go further these series expansions with more specific pdfs. For instance, for the TCPW distribution, owing to (9) and the generalized binomial formula applied to $G_{\alpha (2k+1)}\left(x;\xi \right)$, we get

(11) $$\begin{array}{c} F\left(x;\alpha ,\lambda ,\theta \right)=\sum_{k,\ell =0}^{+\infty }u_{k,\ell }S\left(x;\ell \lambda ,\theta \right), \end{array}$$

where uk,ℓ=(4/π)α(2k+1)ℓ(−1)k+ℓ/(2k+1) and $S\left(x;\ell \lambda ,\theta \right)=e^{-\ell \lambda x^{\theta }}$ which is the survival function of the Weibull distribution with parameters ℓλ and θ. Upon differentiation of F(x;α,λ,θ), we get

(12) $$\begin{array}{c} f\left(x;\alpha ,\lambda ,\theta \right)=\sum_{k=0}^{+\infty }\sum_{\ell =1}^{+\infty }v_{k,\ell }g\left(x;\ell \lambda ,\theta \right), \end{array}$$

where vk,ℓ=−uk,ℓ=(4/π)α(2k+1)ℓ(−1)k+ℓ+1/(2k+1) and $g\left(x;\ell \lambda ,\theta \right)=\ell \lambda \theta x^{\theta -1}e^{-\ell \lambda x^{\theta }}$ which is the pdf of the Weibull distribution with parameters ℓλ and θ.

### 3.2. On Moments and Related Functions

Now, let *X* be a random variable with the cdf given by (1), defined on a probability space (Ω,A,P).

By virtue of (10), for any function ψ(x) such that all the introduced quantities are well-defined, we have the following integral expression:

$$\begin{array}{c} E\left[\psi \left(X\right)\right]=\int_{-\infty }^{+\infty }\psi \left(x\right)f\left(x;\alpha ,\xi \right)dx=\frac{4}{\pi }\sum_{k=0}^{+\infty }\frac{{\left(-1\right)}^{k}}{2k+1}\int_{-\infty }^{+\infty }\psi \left(x\right)g_{\alpha (2k+1)}\left(x;\xi \right)dx. \end{array}$$

For some configurations, the integral term can be calculated or, at least, evaluated numerically by any mathematical software.

In particular, the *s*-th moment of *X* is obtained by choosing $\psi \left(x\right)=x^{s}$, i.e., $\mu_{s}^{\prime }\left(\alpha ,\xi \right)=E\left(X^{s}\right)$. Hence, by taking s=1, we get the mean of *X*, i.e., $\mu \left(\alpha ,\xi \right)=\mu_{1}^{\prime }\left(\alpha ,\xi \right)$. Furthermore, by taking s=2, we obtain $\mu_{2}^{\prime }\left(\alpha ,\xi \right)=E\left(X^{2}\right)$, from which we can express the variance of *X* defined by $\sigma^{2}\left(\alpha ,\xi \right)=\mu_{2}^{\prime }\left(\alpha ,\xi \right)-\mu {\left(\alpha ,\xi \right)}^{2}$. From the first *s* moments of *X*, the *s*-th central moment of *X* can be deduced as

μs(α,ξ)=E[X−μ(α,ξ)]s=∑k=0ssk(−1)kμ(α,ξ)kμs−k′(α,ξ).

Then, some properties of the TCP-G family, as the skewness and kurtosis properties, can be investigated by the study of the *s*-th general coefficient of *X* given by $C_{s}\left(\alpha ,\xi \right)=\mu_{s}\left(\alpha ,\xi \right)/\sigma {\left(\alpha ,\xi \right)}^{s}$.

The moment generation function of *X* according to *t* is obtained by choosing $\psi \left(x\right)=\psi_{t}\left(x\right)=e^{tX}$, i.e., $M\left(t;\alpha ,\xi \right)=E\left(e^{tX}\right)$. Similarly, the characteristic function of *X* according to *t* is obtained by choosing $\psi \left(x\right)=\psi_{it}\left(x\right)=e^{itx}$, where $i^{2}=-1$, i.e., $\varphi \left(t;\alpha ,\xi \right)=E\left(e^{itX}\right)$.

Another important function is the *s*-th incomplete moment of *X* according to *y* which follows from the choice $\psi \left(x\right)=\psi_{y}^{*}\left(x\right)=x^{s}1_{\left\{x\leq y\right\}}$, where $1_{A}$ denotes the indicator function equal to one if *A* is satisfied and 0 otherwise, i.e., $\mu_{s}^{\prime }\left(y;\alpha ,\xi \right)=E\left(X^{s}1_{\left\{X\leq y\right\}}\right)$. In particular, the first incomplete moment allows us to define the mean deviation about the mean, i.e., $\delta_{1}\left(\alpha ,\xi \right)=E[|X-\mu \left(\alpha ,\xi \right)|]$, the mean deviation about the median, i.e., $\delta_{2}\left(\alpha ,\xi \right)=E[|X-M\left(\alpha ,\xi \right)|]$, as well as the Lorenz curve, the Gini inequality index and the Zenga curve, which are of great importance in many applied fields. Further details can be found in [27,28].

Let us now discuss some of the above properties in the context of the TCPW distribution, with the use of (12). Thus, *X* is a random variable following the TCPW distribution, i.e., having the cdf given by (5). Then, the *s*-th moment $\mu_{s}^{\prime }\left(\alpha ,\lambda ,\theta \right)$ exists. Owing to (12) and $\int_{0}^{+\infty }x^{s}g\left(x;\ell \lambda ,\theta \right)dx={\left(\ell \lambda \right)}^{-s/\theta }\Gamma \left(1+s/\theta \right)$, where $\Gamma \left(x\right)=\int_{0}^{+\infty }t^{x-1}e^{-t}dt$, one can express it as

$$\begin{array}{c} \mu_{s}^{\prime }\left(\alpha ,\lambda ,\theta \right)=\lambda^{-s/\theta }\Gamma \left(1+\frac{s}{\theta }\right)\sum_{k=0}^{+\infty }\sum_{\ell =1}^{+\infty }v_{k,\ell }\ell^{-s/\theta }. \end{array}$$

**Figure 3:**
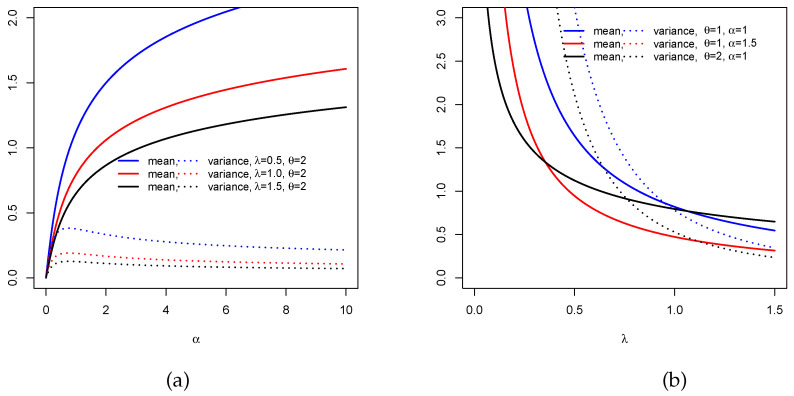
Plots of the mean and variance for the TCPW distribution: (**a**) for fixed λ and θ and varying α and (**b**) for fixed θ and α and varying λ.

That is, we obtain the mean μ(α,λ,θ) and the variance $\sigma^{2}\left(\alpha ,\lambda ,\theta \right)$ of *X* proceeding as above. To illustrate the effect of the parameters α, λ and θ on them, Figure 3 represents μ(α,λ,θ) and $\sigma^{2}\left(\alpha ,\lambda ,\theta \right)$ under two different scenarios: (i) for fixed λ and θ and varying α and (ii) for fixed θ and α and varying λ. Wee that the mean can increase with a near constant variance (see Figure 3a) whereas it can decrease with high variations for the variance (Figure 3b). This illustrates the flexibility of these two measures according to the distribution parameters.

We conclude this part by the description of the incomplete moments of *X*. By introducing the lower incomplete gamma function defined by $\gamma \left(x,y\right)=\int_{0}^{y}t^{x-1}e^{-t}dt$, the *s*-th incomplete moment of *X* is given by

$$\begin{array}{c} \mu_{s}^{\prime }\left(y;\alpha ,\lambda ,\theta \right)=\lambda^{-s/\theta }\sum_{k=0}^{+\infty }\sum_{\ell =1}^{+\infty }v_{k,\ell }\ell^{-s/\theta }\gamma \left(1+\frac{s}{\theta },\ell \lambda y^{\theta }\right). \end{array}$$

Thus, the first incomplete moment can be derived, as well as the related important quantities and functions (mean deviations, Lorenz curve…).

### 3.3. Skewness and Kurtosis Based on Quantiles

As previously mentioned, one can define measures of skewness and kurtosis based on quantiles. In comparison to those defined with moments, they are more simple to calculate and not influenced by the eventual extreme tails of the distribution. One of the most useful skewness based on quantile is the MacGillivray skewness introduced by [29]. In the context of the TCP-G family, based on (4) and the median, it is given by the following function:

$$\rho \left(u;\alpha ,\xi \right)=\frac{Q(1-u;\alpha ,\xi )+Q(u;\alpha ,\xi )-2M(\alpha ,\xi )}{Q(1-u;\alpha ,\xi )-Q(u;\alpha ,\xi )},\;u\in \left(0,1\right).$$

We can use this robust function to describe efficiently the effect of the parameters (α,ξ) on the skewness; more the shapes of the graphs of ρ(u;α,λ,θ) are varying according to the parameters, more the skewness is flexible. One can notice that, for u=1/4, it becomes the Galton skewness studied by [30]. The sign of the Galton skewness is informative on the right or symmetric or left skewed nature of the distribution; ρ(3/4;α,ξ)>0 means that the distribution is right skewed, ρ(3/4;α,ξ)=0 means that the distribution is symmetrical and ρ(3/4;α,ξ)<0 means that the distribution is left skewed.

Also, the kurtosis of the TCP-G family can be studied by considering the Moors kurtosis proposed by [31]. It is defined by

$$K\left(\alpha ,\xi \right)=\frac{Q(7/8;\alpha ,\xi )-Q(5/8;\alpha ,\xi )+Q(3/8;\alpha ,\xi )-Q(1/8;\alpha ,\xi )}{Q(3/4;\alpha ,\xi )-Q(1/4;\alpha ,\xi )}.$$

A high value for K(α,ξ) means that the distribution has heavy tails and a small values for K(α,ξ) means that the distribution has light tails.

**Figure 4:**
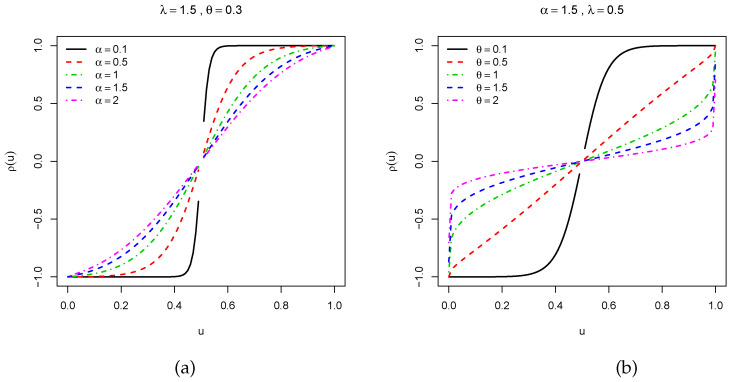
Plots of the MacGillivray skewness for selected values of the parameters when (**a**) α increases and (**b**) θ increases.

**Figure 5:**
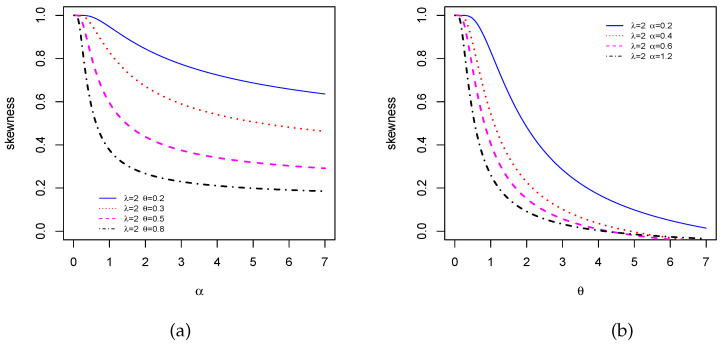
Plots of Galton skewness for selected values of the parameters when (**a**) α varies and (**b**) θ varies.

**Figure 6:**
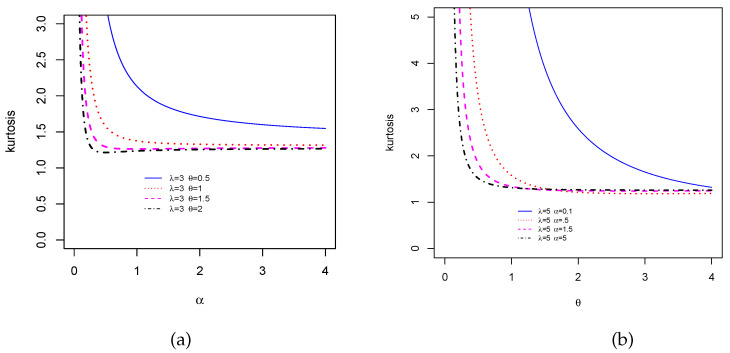
Plots of Moors kurtosis for selected values of the parameters when (**a**) α varies and (**b**) θ varies.

We now investigate the skewness and kurtosis of the TCPW distribution. In this case, thanks to (8), the MacGillivray skewness and Moors kurtosis have a closed-form. We now propose some visual explorations of these measures. Figure 4 presents the MacGillivray skewness when (i) λ and θ are constant, i.e., λ=1.5 and θ=0.3, and α increases and (ii) α and λ are constant, i.e., α=1.5 and λ=0.5, and θ increases. Moderate variations can be seen in the curves of Figure 4a, meaning that the parameter α has a moderate effect on the skewness, whereas various wide variations on the shapes of the curves are observed in Figure 4b, showing that the parameter θ strongly influenced the skewness. Then, a similar visual approach is performed for the Galton skewness in Figure 5. For the selected values of the parameters, we see that the Galton skewness decreases. Also, it is observed that it can be positive (see Figure 5a) or negative (see Figure 5b with λ=2, α∈{0.4,0.6,1.2} and θ>5 approximately), meaning that the TCPW distribution can be left or right skewed, respectively. Figure 6 displays the Moors kurtosis following the same scenarios. We see that the TCPW distribution can be of different kurtosis nature, which small or high possible values. All these facts show the great skewness and kurtosis flexibility of the TCPW distribution.

### 3.4. Rényi Entropy and *q*-Entropy

Entropy is a fundamental measure to quantify the amount of informations in a distribution, finding applications in information science, thermodynamics and statistical physics. Here, we investigate two different and complementary kinds of entropy arising from various physical experiments: Rényi entropy and *q*-entropy, of the TCP-G family, as introduced by [32,33], respectively. As common interpretation, the lower the entropy, the lower the randomness of the related system. For further detail, we refer the reader to the survey of [34].

Rényi entropy is defined by

$$\begin{array}{c} I_{\delta }\left(\alpha ,\xi \right)=\frac{1}{1-\delta }\log\left[\int_{-\infty }^{+\infty }f{\left(x;\alpha ,\xi \right)}^{\delta }dx\right], \end{array}$$

with δ∈(0,+∞){1}. Since it can be expressed analytically, we aims to provide a series expansion of $I_{\delta }\left(\alpha ,\xi \right)$. Owing to (2) and the generalized binomial formula, we get

f(x;α,ξ)δ=4δαδπδ∑k=0+∞−δkg(x;ξ)δG2αk+δ(α−1)(x;ξ).

Therefore, we can expressed $I_{\delta }\left(\alpha ,\xi \right)$ as:

Iδ(α,ξ)=11−δδlog(4)+δlog(α)−δlog(π)+log∑k=0+∞−δk∫−∞+∞g(x;ξ)δG2αk+δ(α−1)(x;ξ)dx.

For given functions and parameters, mathematical software can be useful to evaluated numerically this last integral.

If we consider the case of the TCPW distribution, we can formulate $I_{\delta }\left(\alpha ,\lambda ,\theta \right)$ by the above expression and the following series expansion:

(13) ∫−∞+∞g(x;λ,θ)δG2αk+δ(α−1)(x;λ,θ)dx=λ(δ−1)/θθδ−1∑k=0+∞2αk+δ(α−1)k(−1)k(δ+k)−1−(1−1/θ)(δ−1)Γ1+(θ−1)(δ−1)θ.

In the general context of the TCP-G family, the *q*-entropy is defined by

$$\begin{array}{c} H_{q}\left(\alpha ,\xi \right)=\frac{1}{1-q}\left[1-\int_{-\infty }^{+\infty }f{\left(x;\alpha ,\xi \right)}^{q}dx\right], \end{array}$$

with δ∈(0,+∞){1}. Proceeding as for the Rényi entropy, we can expressed it as:

Hq(α,ξ)=11−q1−4qαqπq∑k=0+∞−qk∫−∞+∞g(x;ξ)qG2αk+q(α−1)(x;ξ)dx.

For the the TCPW distribution, by replacing δ by *q*, we can express the integral term as in (13).

### 3.5. Order Statistics

We now present the main properties of the order statistics in the context of the TCP-G family. The general theory can be found in [35].

Now, let $X_{1},\ldots ,X_{n}$ be a random sample from the TCP-G family and $X_{i:n}$ be the *i*-th order statistic, i.e., its *i*-th smallest random variables (in the standard probabilistic ordering sense, i.e., X≤Y if and only if P(X≤Y)=1). Then, it is well-known that the cdf and pdf of $X_{i:n}$ are, respectively, given by

Fi:n(x;α,ξ)=n!(i−1)!(n−i)!∑j=0n−in−ij(−1)j1j+iF(x;α,ξ)j+i,x∈R

and

(14) $$\begin{array}{c} f_{i:n}\left(x;\alpha ,\xi \right)=\frac{n!}{(i-1)!(n-i)!}F{\left(x;\alpha ,\xi \right)}^{i-1}{\left[1-F\left(x;\alpha ,\xi \right)\right]}^{n-i}f\left(x;\alpha ,\xi \right),\;x\in R. \end{array}$$

We now focus on the determination of a tractable series expansions for $F_{i:n}\left(x;\alpha ,\xi \right)$ and $f_{i:n}\left(x;\alpha ,\xi \right)$. In this regard, let us now present a result on the series expansion for the exponentiated arctangent function with power integer. For any x∈[−1,1] and any integer *s*, we have

(15) $$\begin{array}{c} {\left[\arctan\left(x\right)\right]}^{s}=\sum_{k=0}^{+\infty }c_{s,k}x^{2k+s}, \end{array}$$

where $c_{s,0}=1$ and, for any m≥1, $c_{s,m}$ is defined by the following relation:

$$c_{s,m}=\frac{1}{m}\sum_{\ell =1}^{m}\left[\ell \left(s+1\right)-m\right]\frac{{\left(-1\right)}^{\ell }}{2\ell +1}c_{s,m-\ell },$$

(thus, for instance, $c_{s,1}=-s/3$ and $c_{s,2}=s^{2}/18+13s/90$). The proof of this intermediary result is discussed below. Owing to [26] (Point 0.314), for an integer *s*, a sequence of real numbers ${\left(a_{k}\right)}_{k\in N}$ and y∈R, by assuming that the introduced sums converge, we have ${\left(\sum_{k=0}^{+\infty }a_{k}y^{k}\right)}^{s}=\sum_{k=0}^{+\infty }c_{s,k}y^{k}$, where the coefficients ${\left(c_{s,k}\right)}_{k\in N}$ are determined by the following relations: $c_{s,0}=a_{0}^{s}$ and, for any m≥1, $c_{s,m}={\left(ma_{0}\right)}^{-1}\sum_{\ell =1}^{m}\left[\ell \left(s+1\right)-m\right]a_{\ell }c_{s,m-\ell }$. Since, for any x∈[−1,1], we have $\arctan\left(x\right)=\sum_{k=0}^{+\infty }a_{k}x^{2k+1}$, with $a_{k}={\left(-1\right)}^{k}/\left(2k+1\right)$ (so $a_{0}=1$), the above result implies that

$$\begin{array}{c} {\left[\arctan\left(x\right)\right]}^{s}=x^{s}{\left(\sum_{k=0}^{+\infty }a_{k}{\left(x^{2}\right)}^{k}\right)}^{s}=x^{s}\sum_{k=0}^{+\infty }c_{s,k}{\left(x^{2}\right)}^{k}=\sum_{k=0}^{+\infty }c_{s,k}x^{2k+s}. \end{array}$$

Thus, it follows from (15) that

Fi:n(x;α,ξ)=n!(i−1)!(n−i)!∑j=0n−in−ij(−1)j1j+i4πarctan[G(x;ξ)α]j+i=∑j=0n−i∑k=0+∞dj,k;i:nGα(2k+j+i)(x;ξ),

where

di,j;i:n=n!(i−1)!(n−i)!n−ij(−1)j1j+i4πj+icj+i,k,

($c_{j+i,k}$ is defined as in (15) with s=j+i). This shows that the cdf of the order statistics of the TCP-G family can be expressed as an infinite mixture of cdfs of the exponentiated-G family by [8]. Therefore, the well-established properties of the exponentiated-G family can be used to determine those of the order statistics of the TCP-G family. Indeed, from $F_{i:n}\left(x;\alpha ,\xi \right)$, one can deduce the corresponding pdf by differentiation as follows:

$$\begin{array}{c} f_{i:n}\left(x;\alpha ,\xi \right)=\sum_{j=0}^{n-i}\sum_{k=0}^{+\infty }d_{j,k;i:n}g_{\alpha (2k+j+i)}\left(x;\xi \right). \end{array}$$

This expression allows determining moments, skewness, kurtosis, and other important measures and functions.

In the case of the TCPW distribution, a refinement of these series expansions are possible. Indeed, we can expend $G_{\alpha (2k+j+i)}\left(x;\xi \right)$ in a series expansion as in (11), which implies that

$$\begin{array}{c} F_{i:n}\left(x;\alpha ,\lambda ,\theta \right)=\sum_{j=0}^{n-i}\sum_{k,\ell =0}^{+\infty }e_{j,k,\ell ;i:n}S\left(x;\ell \lambda ,\theta \right), \end{array}$$

where ej,k,ℓ;i:n=α(2k+j+i)ℓ(−1)ℓdi,j;i:n and $S\left(x;\ell \lambda ,\theta \right)=e^{-\ell \lambda x^{\theta }}$ (we recall that it is the survival function of the Weibull distribution with parameters ℓλ and θ).

Also, upon differentiation of $F_{i:n}\left(x;\alpha ,\lambda ,\theta \right)$, the pdf of $X_{i:n}$ is given by

$$\begin{array}{c} f_{i:n}\left(x;\alpha ,\lambda ,\theta \right)=\sum_{j=0}^{n-i}\sum_{k=0}^{+\infty }\sum_{\ell =1}^{+\infty }e_{j,k,\ell ;i:n}g\left(x;\ell \lambda ,\theta \right), \end{array}$$

where $g\left(x;\ell \lambda ,\theta \right)=\ell \lambda \theta x^{\theta -1}e^{-\ell \lambda x^{\theta }}$ (we recall that it is the pdf of the Weibull distribution with parameters ℓλ and θ). As a direct application, the *r*-th moment of $X_{i:n}$ can be obtained as

$$\begin{array}{cc} \mu_{r,i:n}^{\prime }\left(\alpha ,\lambda ,\theta \right) & =E\left(X_{i:n}^{r}\right)=\lambda^{-s/\theta }\Gamma \left(1+\frac{s}{\theta }\right)\sum_{j=0}^{n-i}\sum_{k=0}^{+\infty }\sum_{\ell =1}^{+\infty }e_{j,k,\ell ;i:n}\ell^{-s/\theta }. \end{array}$$

## 4. Estimation of the TCP-G Model Parameters

This section is devoted to the inferential properties of the TCP-G model. The estimation of the parameters α and ξ is performed by the maximum likelihood method. Two different sampling schemes are considered: the simple random sampling (SRS) and the ranked set sampling (RSS). In what follows, *n* denotes a positive integer measuring the size of the considered sample; it can be small or large.

### 4.1. Maximum Likelihood Method under SRS

Let $x_{1},\ldots ,x_{n}$ be a SRS from the TCP-G family, i.e., with the pdf given by (2). Then, the corresponding likelihood function is defined by

$$L\left(\alpha ,\xi \right)=\prod_{i=1}^{n}f\left(x_{i};\alpha ,\xi \right)=\frac{4^{n}\alpha^{n}}{\pi^{n}}\prod_{i=1}^{n}\frac{g\left(x_{i};\xi \right)G{\left(x_{i};\xi \right)}^{\alpha -1}}{1+G{\left(x_{i};\xi \right)}^{2\alpha }}.$$

Thus, the corresponding log-likelihood function is defined by

$$\begin{array}{cc} \ell (\alpha ,\xi ) & =\log[L(\alpha ,\xi )]=n\log(4)+n\log(\alpha )-n\log(\pi )\\ \; & +\sum_{i=1}^{n}\log\left[g\left(x_{i};\xi \right)\right]+\left(\alpha -1\right)\sum_{i=1}^{n}\log\left[G\left(x_{i};\xi \right)\right]-\sum_{i=1}^{n}\log\left[1+G{\left(x_{i};\xi \right)}^{2\alpha }\right]. \end{array}$$

Then, the maximum likelihood estimates (MLEs) of α and ξ are defined by $\left(\hat{\alpha },\hat{\xi }\right)={\mathrm{argmax}}_{\left(\alpha ,\xi \right)}L\left(\alpha ,\xi \right)={\mathrm{argmax}}_{\left(\alpha ,\xi \right)}\ell \left(\alpha ,\xi \right)$. Assuming that ℓ(α,ξ) is differentiable, the MLEs can be obtained by solving the following non-linear equations simultaneously: $\partial \ell (\hat{\alpha },\hat{\xi })/\partial \alpha =0$ and $\partial \ell (\hat{\alpha },\hat{\xi })/\partial \xi =0$, with

$$\begin{array}{c} \frac{\partial \ell (\alpha ,\xi )}{\partial \alpha }=\frac{n}{\alpha }+\sum_{i=1}^{n}\log\left[G\left(x_{i};\xi \right)\right]-2\sum_{i=1}^{n}\frac{G{\left(x_{i};\xi \right)}^{2\alpha }\log\left[G\left(x_{i};\xi \right)\right]}{1+G{\left(x_{i};\xi \right)}^{2\alpha }} \end{array}$$

and, by setting $g{\left(x_{i};\xi \right)}_{\xi }=\partial g\left(x_{i};\xi \right)/\partial \xi$ and $G{\left(x_{i};\xi \right)}_{\xi }=\partial G\left(x_{i};\xi \right)/\partial \xi$,

$$\begin{array}{c} \frac{\partial \ell (\alpha ,\xi )}{\partial \xi }=\sum_{i=1}^{n}\frac{g{\left(x_{i};\xi \right)}_{\xi }}{g(x_{i};\xi )}+\left(\alpha -1\right)\sum_{i=1}^{n}\frac{G{\left(x_{i};\xi \right)}_{\xi }}{G(x_{i};\xi )}-2\alpha \sum_{i=1}^{n}\frac{G{\left(x_{i};\xi \right)}_{\xi }G{\left(x_{i};\xi \right)}^{2\alpha -1}}{1+G{\left(x_{i};\xi \right)}^{2\alpha }}. \end{array}$$

In general, these non-linear equations cannot be solved explicitly. However, the corresponding MLEs can be evaluated by using any well-know numerical numerical optimization technique. Thanks to the well-established theory of the maximum likelihood maximum method, by assuming that *n* is large enough and some regularity conditions hold, we can construct asymptotic confidence intervals of the model parameters. In this regard, we need the approximate inverse of the observed information matrix. By setting *r* be the number of components in the vector ξ and $\xi =(\xi_{1},\ldots ,\xi_{r})$, it is given by

(16) $$\begin{array}{c} I{\left(\hat{\alpha },\hat{\xi }\right)}^{-1}=-{\left.{\left(\begin{array}{ccccc} \frac{\partial^{2}\ell \left(\alpha ,\xi \right)}{\partial \alpha^{2}} & \frac{\partial^{2}\ell \left(\alpha ,\xi \right)}{\partial \alpha \partial \xi_{1}} & \frac{\partial^{2}\ell \left(\alpha ,\xi \right)}{\partial \alpha \partial \xi_{2}} & \cdots & \frac{\partial^{2}\ell \left(\alpha ,\xi \right)}{\partial \alpha \partial \xi_{r}}\\ . & \frac{\partial^{2}\ell \left(\alpha ,\xi \right)}{\partial \xi_{1}^{2}} & \frac{\partial^{2}\ell \left(\alpha ,\xi \right)}{\partial \xi_{1}\partial \xi_{2}} & \cdots & \frac{\partial^{2}\ell \left(\alpha ,\xi \right)}{\partial \xi_{1}\partial \xi_{r}}\\ . & . & \frac{\partial^{2}\ell \left(\alpha ,\xi \right)}{\partial \xi_{2}^{2}} & \cdots & \frac{\partial^{2}\ell \left(\alpha ,\xi \right)}{\partial \xi_{2}\partial \xi_{r}}\\ . & . & . & \cdots & .\\ . & . & . & \cdots & .\\ . & . & . & \cdots & \frac{\partial^{2}\ell \left(\alpha ,\xi \right)}{\partial \xi_{r}^{2}} \end{array}\right)}^{-1}\right|}_{\left(\alpha ,\xi \right)=\left(\hat{\alpha },\hat{\xi }\right)}. \end{array}$$

Then, the asymptotic confidence intervals of α and $\xi_{i}$, for i=1,…,r, at the level 100(1−ν)% are, respectively, given by

(17) $$\begin{array}{c} CI_{\alpha }=\left[\hat{\alpha }-z_{1-\nu /2}\sqrt{v_{\hat{\alpha }}},\hat{\alpha }+z_{1-\nu /2}\sqrt{v_{\hat{\alpha }}}\right],\;CI_{\xi_{i}}=\left[{\hat{\xi }}_{i}-z_{1-\nu /2}\sqrt{v_{{\hat{\xi }}_{i}}},{\hat{\xi }}_{i}+z_{1-\nu /2}\sqrt{v_{{\hat{\xi }}_{i}}}\right], \end{array}$$

where $v_{\hat{\alpha }}$ and $v_{{\hat{\xi }}_{i}}$ are the first and i+1-th elements of the main diagonal of $I{\left(\hat{\alpha },\hat{\xi }\right)}^{-1}$, respectively, and $z_{\gamma }$ is the quantile of the standard normal distribution taken at γ.

For the special case of the TCPW model, we recall that ξ=(λ,θ), $G\left(x;\lambda ,\theta \right)=1-e^{-\lambda x^{\theta }}$ and $g\left(x;\lambda ,\theta \right)=\lambda \theta x^{\theta -1}e^{-\lambda x^{\theta }}$. Thus, the equations to obtain the MLEs $\hat{\alpha }$, $\hat{\lambda }$ and $\hat{\theta }$ of α, λ and θ, respectively, can be expressed by using the following partial derivatives:

$$\begin{array}{c} G{\left(x;\lambda ,\theta \right)}_{\lambda }=x^{\theta }e^{-\lambda x^{\theta }},\;G{\left(x;\lambda ,\theta \right)}_{\theta }=\lambda x^{\theta }\log\left(x\right)e^{-\lambda x^{\theta }}, \end{array}$$

$$\begin{array}{c} g{\left(x;\lambda ,\theta \right)}_{\lambda }=\theta x^{\theta -1}\left(1-\lambda x^{\theta }\right)e^{-\lambda x^{\theta }},\;g{\left(x;\lambda ,\theta \right)}_{\theta }=\theta x^{\theta -1}\left[1+\theta \log\left(x\right)-\lambda \theta x^{\theta }\log\left(x\right)\right]e^{-\lambda x^{\theta }}. \end{array}$$

The same for the approximate inverse of the observed information matrix, i.e., $I{\left(\hat{\alpha },\hat{\lambda },\hat{\theta }\right)}^{-1}$, but with the determination of the second partial derivatives. Here, we omit them for the sake of place.

### 4.2. Maximum Likelihood Method under RSS

First of all, let us briefly present the considered RSS as introduced by [36] in our distributional context and in the following simple scheme: it is supposed that the set size is *n* and that number of cycles is *n*. In this scheme, let $x_{1},\ldots ,x_{n^{2}}$ be a SRS of size $n^{2}$ from the TCP-G family, i.e., with the cdf and pdf given by (1) and (2). Then, the obtained values are randomly divided into *n* sets of *n* units each. On each set, we rank the *n* elements. In the first set, we select the element with the smallest ranking, denoted by $x_{1(1)}$. In the second set, we select the element with the second smallest ranking, denoted by $x_{2(2)}$. We follow this processes until we have ranked the elements in the *n*-th set and selected the element with the largest ranking, denoted by $x_{n(n)}$.

Adopting the framework above, the corresponding likelihood function is defined by

$$\begin{array}{cc} \; & L_{*}\left(\alpha ,\xi \right)=\prod_{i=1}^{n}f_{i:n}\left(x_{i(i)};\alpha ,\xi \right)=\tau_{n}\prod_{i=1}^{n}\left\{F{\left(x_{i(i)};\alpha ,\xi \right)}^{i-1}{\left[1-F\left(x_{i(i)};\alpha ,\xi \right)\right]}^{n-i}f\left(x_{i(i)};\alpha ,\xi \right)\right\}\\ \; & =\tau_{n}\frac{4^{n}\alpha^{n}}{\pi^{n}}\prod_{i=1}^{n}\left\{{\left\{\frac{4}{\pi }\arctan\left[G{\left(x_{i(i)};\xi \right)}^{\alpha }\right]\right\}}^{i-1}{\left[1-\frac{4}{\pi }\arctan\left[G{\left(x_{i(i)};\xi \right)}^{\alpha }\right]\right]}^{n-i}\frac{g\left(x_{i(i)};\xi \right)G{\left(x_{i(i)};\xi \right)}^{\alpha -1}}{1+G{\left(x_{i(i)};\xi \right)}^{2\alpha }}\right\}, \end{array}$$

where $\tau_{n}=\prod_{i=1}^{n}n!/\left[\left(i-1\right)!\left(n-i\right)!\right]$.

Thus, the corresponding log-likelihood function is defined by

$$\begin{array}{cc} \ell_{*}\left(\alpha ,\xi \right) & =\log\left[L_{*}\left(\alpha ,\xi \right)\right]=\log\left(\tau_{n}\right)+n\log\left(4\right)+n\log\left(\alpha \right)-n\log\left(\pi \right)\\ \; & +\sum_{i=1}^{n}\left(i-1\right)\log\left\{\frac{4}{\pi }\arctan\left[G{\left(x_{i(i)};\xi \right)}^{\alpha }\right]\right\}+\sum_{i=1}^{n}\left(n-i\right)\log\left\{1-\frac{4}{\pi }\arctan\left[G{\left(x_{i(i)};\xi \right)}^{\alpha }\right]\right\}\\ \; & +\sum_{i=1}^{n}\log\left[g\left(x_{i(i)};\xi \right)\right]+\left(\alpha -1\right)\sum_{i=1}^{n}\log\left[G\left(x_{i(i)};\xi \right)\right]-\sum_{i=1}^{n}\log\left[1+G{\left(x_{i(i)};\xi \right)}^{2\alpha }\right]. \end{array}$$

Then, the maximum likelihood estimates (MLEs) of α and ξ are defined by $\left(\tilde{\alpha },\tilde{\xi }\right)={\mathrm{argmax}}_{\left(\alpha ,\xi \right)}L_{*}\left(\alpha ,\xi \right)={\mathrm{argmax}}_{\left(\alpha ,\xi \right)}\ell_{*}\left(\alpha ,\xi \right)$. Assuming that $\ell_{*}\left(\alpha ,\xi \right)$ is differentiable, the MLEs can be obtained by solving the following non-linear equations simultaneously: $\partial \ell_{*}\left(\tilde{\alpha },\tilde{\xi }\right)/\partial \alpha =0$ and $\partial \ell_{*}\left(\tilde{\alpha },\tilde{\xi }\right)/\partial \xi =0$, with

$$\begin{array}{cc} \frac{\partial \ell_{*}\left(\alpha ,\xi \right)}{\partial \alpha } & =\frac{n}{\alpha }+\sum_{i=1}^{n}\left(i-1\right)\frac{G{\left(x_{i(i)};\xi \right)}^{\alpha }\log\left[G\left(x_{i(i)};\xi \right)\right]}{\left[1+G{\left(x_{i(i)};\xi \right)}^{2\alpha }\right]\arctan\left[G{\left(x_{i(i)};\xi \right)}^{\alpha }\right]}\\ \; & -\frac{4}{\pi }\sum_{i=1}^{n}\left(n-i\right)\frac{G{\left(x_{i(i)};\xi \right)}^{\alpha }\log\left[G\left(x_{i(i)};\xi \right)\right]}{\left[1+G{\left(x_{i(i)};\xi \right)}^{2\alpha }\right]\left\{1-\left(4/\pi \right)\arctan[G{\left(x_{i(i)};\xi \right)}^{\alpha }]\right\}}\\ \; & +\sum_{i=1}^{n}\log\left[G\left(x_{i(i)};\xi \right)\right]-2\sum_{i=1}^{n}\frac{G{\left(x_{i(i)};\xi \right)}^{2\alpha }\log\left[G\left(x_{i(i)};\xi \right)\right]}{1+G{\left(x_{i(i)};\xi \right)}^{2\alpha }}. \end{array}$$

and, by setting $g{\left(x_{i(i)};\xi \right)}_{\xi }=\partial g\left(x_{i(i)};\xi \right)/\partial \xi$ and $G{\left(x_{i(i)};\xi \right)}_{\xi }=\partial G\left(x_{i(i)};\xi \right)/\partial \xi$,

$$\begin{array}{cc} \frac{\partial \ell_{*}\left(\alpha ,\xi \right)}{\partial \xi } & =\alpha \sum_{i=1}^{n}\left(i-1\right)\frac{g{\left(x_{i(i)};\xi \right)}_{\xi }G{\left(x_{i(i)};\xi \right)}^{\alpha -1}}{\left[1+G{\left(x_{i(i)};\xi \right)}^{2\alpha }\right]\arctan\left[G{\left(x_{i(i)};\xi \right)}^{\alpha }\right]}\\ \; & -\frac{4}{\pi }\alpha \sum_{i=1}^{n}\left(n-i\right)\frac{g{\left(x_{i(i)};\xi \right)}_{\xi }G{\left(x_{i(i)};\xi \right)}^{\alpha -1}}{\left[1+G{\left(x_{i(i)};\xi \right)}^{2\alpha }\right]\left\{1-\left(4/\pi \right)\arctan[G{\left(x_{i(i)};\xi \right)}^{\alpha }]\right\}}\\ \; & +\sum_{i=1}^{n}\frac{g{\left(x_{i(i)};\xi \right)}_{\xi }}{g(x_{i(i)};\xi )}+\left(\alpha -1\right)\sum_{i=1}^{n}\frac{G{\left(x_{i(i)};\xi \right)}_{\xi }}{G(x_{i(i)};\xi )}-2\alpha \sum_{i=1}^{n}\frac{G{\left(x_{i(i)};\xi \right)}_{\xi }G{\left(x_{i(i)};\xi \right)}^{2\alpha -1}}{1+G{\left(x_{i(i)};\xi \right)}^{2\alpha }}. \end{array}$$

In general, these non-linear equations cannot be solved explicitly, but the corresponding MLEs can be obtained by using appropriated numerical technique. Also, the well-known theory of the maximum likelihood method can be applied. In particular, one can construct asymptotic confidence intervals of the model parameters as for the SRS case. In this regard, we need to defined the inverse of the observed information matrix as (16) but with $\ell_{*}\left(\alpha ,\xi \right)$ instead of ℓ(α,ξ) and $\left(\tilde{\alpha },\tilde{\xi }\right)$ instead of $\left(\hat{\alpha },\hat{\xi }\right)$, then the definition of the asymptotic confidence intervals are similar to those in (17) with this new configuration. For the TCPW model, some of the quantities above can be expressed in a similar way to the SRS case.

### 4.3. Simulation Study

As a logical sequel of the previous subsection, we provide a numerical study on the MLEs of the TCPW model parameters based on simple random sampling (SRS) and ranked set sampling (RSS). A comparison study between the estimates is performed by considering the mean squared errors (MSEs) and relative efficients (REs) defined by RE = MSE(RSS)/MSE(SRS). Also, lower bounds (LBs), upper bounds (UBs) of the related asymptotic confidence intervals, as well as their average lengths (ALs) defined by AL = UB - LB at the levels 90% and 95%, are calculated based on RSS and SRS via Mathematica 9. The simulation procedure follows the following six steps.

Step 1: We consider n=100, 200 and 300.
Step 2: The parameters values are selected as
  Set1: (α=0.5,λ=1.5,θ=0.5),
  Set2: (α=1.2,λ=1.5,θ=0.5),
  Set3: (α=1.2,λ=1.5,θ=0.75),
  Set4: (α=0.5,λ=1.5,θ=0.75).
Step 3: For the chosen set of parameters and each sample of size *n*, the MLEs are computed under SRS and RSS as described in the above subsection.
Step 4: Repeat the previous steps from 1 to 3, *N* times representing with different samples, where N=1000. Then, MSEs and REs are computed.
Step 5: The LB, UB and AL for selected values of parameters are calculated based on SRS and RSS.
Step 6: Numerical outcomes are given in Table 1, Table 2, Table 3, Table 4, Table 5, Table 6, Table 7 and Table 8.

From Table 1, Table 2, Table 3, Table 4, Table 5, Table 6, Table 7 and Table 8, for most of the situations, the following comments can be formulated.

- For both of the sampling schemes, the MSEs decrease as *n* increases.
- For both of the sampling schemes, the AL of the CI become decreases as *n* increases.
- The estimates based on RSS have smaller MSE than the corresponding based on SRS. For this reason, in case of a high level of precision is required, RSS is preferable.

## 5. Application to Two Practical Data Sets

The TCPW model finds a concrete interest in the precise modelling of real life data sets. Here, we illustrate this aspect by considering the two following data sets.

The first data set is taken from tests on the endurance of deep-groove ball bearings. The measurements represent the number of millions revolutions reached by each bearing before fatigue failure (see [37]). The first data set is given by: 17.88, 45.60, 54.12, 68.88, 105.84, 28.92, 48.40, 55.56, 84.12, 127.92, 33.00, 51.84, 67.80, 93.12, 128.04, 41.52, 51.96, 68.64, 98.64, 173.40, 42.12, 54.12, 68.64, 105.12. A basic statistical description of this data set is proposed in Table 9.

From Table 10, we observe that the data are right skewed with a moderate kurtosis, which corresponds to a case covered by the TCPW model.

The second data set refers to a lifetime data set taken from [38] (p 105). The data are: 1.1, 1.4, 1.3, 1.7, 1.9, 1.8, 1.6, 2.2, 1.7, 2.7, 4.1, 1.8, 1.5, 1.2, 1.4, 3, 1.7, 2.3, 1.6, 2. A first statistical description of this data set is presented in Table 10.

From Table 10, we see that the data are highly right skewed with a consequent kurtosis, which is a case also covered by the TCPW model.

Then, we compare the TCPW model to the following well-established models: the Kumaraswamy–Weibull-exponential (KwWE) model by [39], the Kumaraswamy–Weibull (Kw-W) model by [9], the beta Weibull (BW) model by [40], and the standard Weibull (W) model. The results are obtained using the R software.

By respecting the standard in the field, all the parameters will be estimated by the MLEs in the SRS case, even if the simulation study is favorable to the RSS for the TCPW model (see the subsection above). Then, standard measures are taken into account, namely: the Cramér-Von Mises (CVM) statistic, the Anderson-Darling (AD) statistic and the Kolmogorov-Smirnov (KS) statistic along with the corresponding *p*-value. The obtained results are summarized in Table 11 and Table 12 for the first and second data sets, respectively. We see that the TCPW model has the smallest CVM, AD, KS and the greatest *p*-value (with *p*-value ≈0.94 and ≈0.97 for the first and second data sets, respectively, which are quite close to the limit 1), attesting that it is the best model for these data sets.

**Figure 7:**
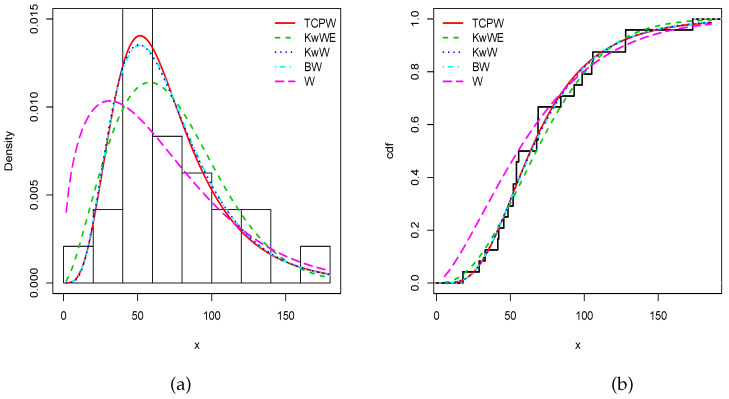
Estimated (**a**) pdfs and (**b**) cdfs of the considered models for the first data set.

**Figure 8:**
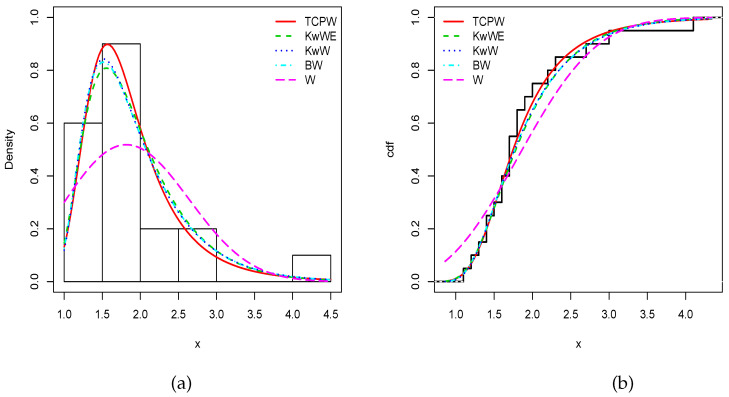
Estimated (**a**) pdfs and (**b**) cdfs of the considered models for the second data set.

To solidify this claim, we provide the minus estimated log-likelihood function ($-\hat{\ell }$), Akaike information criterion (AIC), corrected Akaike information criterion (CAIC), Bayesian information criterion (BIC), and Hannan–Quinn information criterion (HQIC) in Table 13 and Table 14 for the first and second data sets, respectively. We observe that the TCPW model has the smallest AIC, CAIC, BIC and HQIC, attesting its superiority in terms of modelling. To illustrate this, Figure 7 and Figure 8 show the fits of (i) the estimated pdfs over the corresponding histograms and (ii) cdfs over the corresponding empirical cdfs of the related models, for the first and second data sets, respectively. As expected, nice fits can be seen for the TCPW model.

## 6. Concluding Remarks and Perspectives

In this paper, we offered a new general family of distributions based on the truncated Cauchy distribution and the exp-G family, called the truncated Cauchy power-G (TCP-G) family. A focus was put on the special member of the family defined with the Weibull distribution as baseline, called the TCPW distribution. Its cdf has the feature of being simply defined with the arctangent and power functions, allowing tractable expressions for the other corresponding functions (pdf, hrf, qf…). In addition to its simplicity, we revealed the desirable properties of the family, such as very flexible shapes for the pdf and hrf, skewness, kurtosis, moments, entropy…. By considering the special TCPW model, a full simulation study illustrates the nice performance of the maximum likelihood method in the estimation of the model parameters. The deep analysis of two famous data sets shows all the potential of the new family, with fair and favorable comparison to well-established models in the same setting.

From the perspective of this work, one can apply the TCP-G family in a regression model framework (creating new possible distributions on the error term). Also, one can investigate some natural (and not too complicated) extensions of the TCP-G family as those defined by

- the cdf given by
  $$\begin{array}{c} F\left(x;\alpha ,\beta ,\xi \right)={\left\{\frac{4}{\pi }\arctan\left[G{\left(x;\xi \right)}^{\alpha }\right]\right\}}^{\beta },\;x\in R, \end{array}$$
  where α,β>0, which corresponds to the exponentiated cdf of the TCP-G family,
- the cdf given by
  $$\begin{array}{c} F\left(x;\alpha ,\lambda ,\xi \right)=\frac{1}{\arctan(\lambda )}\arctan\left[\lambda G{\left(x;\xi \right)}^{\alpha }\right],\;x\in R, \end{array}$$
  where α>0, λ∈(0,1] and G(x;ξ) denotes the cdf of a univariate continuous distributions with parameter vector denoted by ξ.

These extensions needs further investigations; there is no guarantee as to their superior efficiency over the former TCP-G family is provided at this stage, opening new work chapters for the future.

## Figures and Tables

**Table 1:** Estimates, mean squared errors (MSEs) and relative efficients (REs) for Set1: (α=0.5,λ=1.5,θ=0.5).

*n*	SRS		RSS		RE
	MLE	MSE	MLE	MSE	
100	0.508	0.033	0.512	0.023	0.697
	1.443	0.140	1.509	0.035	0.247
	0.564	0.044	0.524	0.012	0.266
200	0.499	0.024	0.447	0.004	0.173
	1.501	0.110	1.441	0.008	0.072
	0.539	0.022	0.552	0.004	0.181
300	0.492	0.020	0.521	0.002	0.091
	1.519	0.102	1.527	0.003	0.032
	0.533	0.016	0.486	0.001	0.052

**Table 2:** Estimates, mean squared errors (MSEs) and relative efficients (REs) for Set2: (α=1.2,λ=1.5,θ=0.5).

*n*	SRS		RSS		RE
	MLE	MSE	MLE	MSE	
100	1.846	2.534	1.115	0.047	0.019
	1.747	0.645	1.428	0.028	0.044
	0.481	0.027	0.541	0.005	0.198
200	1.201	0.158	1.225	0.030	0.190
	1.371	0.123	1.509	0.016	0.128
	0.519	0.008	0.500	0.002	0.242
300	1.179	0.054	1.224	0.012	0.215
	1.449	0.034	1.517	0.007	0.203
	0.521	0.004	0.498	0.001	0.151

**Table 3:** Estimates, mean squared errors (MSEs) and relative efficients (REs) for Set3: (α=1.2,λ=1.5,θ=0.75).

*n*	SRS		RSS		RE
	MLE	MSE	MLE	MSE	
100	1.253	0.761	1.326	0.154	0.202
	1.412	0.373	1.562	0.060	0.161
	0.945	0.188	0.740	0.014	0.072
200	1.271	0.268	1.209	0.022	0.084
	1.547	0.186	1.501	0.012	0.064
	0.800	0.043	0.752	0.003	0.069
300	1.148	0.091	1.118	0.010	0.115
	1.426	0.047	1.431	0.007	0.137
	0.787	0.015	0.780	0.002	0.117

**Table 4:** Estimates, mean squared errors (MSEs) and relative efficients (REs) for Set4: (α=0.5,λ=1.5,θ=0.75).

*n*	SRS		RSS		RE
	MLE	MSE	MLE	MSE	
100	0.576	0.078	0.475	0.018	0.236
	1.599	0.239	1.464	0.040	0.167
	0.848	0.108	0.843	0.051	0.470
200	0.481	0.024	0.504	0.004	0.182
	1.533	0.100	1.490	0.009	0.090
	0.859	0.057	0.751	0.004	0.069
300	0.472	0.013	0.496	0.001	0.059
	1.473	0.043	1.486	0.001	0.033
	0.795	0.026	0.754	0.001	0.037

**Table 5:** Lower bounds (LBs), upper bounds (UBs), and average lengths (ALs) based on simple random sampling (SRS) and ranked set sampling (RSS) for Set1: (α=0.5,λ=1.5,θ=0.5).

*n*	SRS						RSS					
	90%			95%			90%			95%		
	LB	UB	AL	LB	UB	AL	LB	UB	AL	LB	UB	AL
100	0.124	0.892	0.768	0.050	0.965	0.915	0.141	0.882	0.742	0.070	0.953	0.884
	0.649	2.237	1.588	0.497	2.389	1.892	0.733	2.284	1.551	0.585	2.433	1.848
	0.205	0.923	0.718	0.136	0.992	0.856	0.233	0.816	0.583	0.177	0.871	0.695
200	0.228	0.770	0.542	0.177	0.822	0.646	0.219	0.676	0.457	0.175	0.720	0.545
	0.939	2.064	1.125	0.831	2.172	1.341	0.897	1.984	1.087	0.793	2.088	1.295
	0.317	0.761	0.444	0.274	0.804	0.530	0.338	0.766	0.429	0.297	0.807	0.511
300	0.284	0.700	0.416	0.244	0.740	0.496	0.313	0.729	0.416	0.273	0.769	0.496
	1.073	1.965	0.892	0.987	2.051	1.063	1.098	1.955	0.858	1.016	2.037	1.022
	0.362	0.704	0.341	0.330	0.736	0.407	0.344	0.627	0.284	0.317	0.654	0.338

**Table 6:** Lower bounds (LBs), upper bounds (UBs), and average lengths (ALs) based on simple random sampling (SRS) and ranked set sampling (RSS) for Set2: (α=1.2,λ=1.5,θ=0.5).

*n*	SRS						RSS					
	90%			95%			90%			95%		
	LB	UB	AL	LB	UB	AL	LB	UB	AL	LB	UB	AL
100	−0.097	3.789	3.886	−0.469	4.161	4.630	0.172	2.058	1.886	−0.008	2.239	2.247
	0.848	2.646	1.798	0.676	2.818	2.142	0.600	2.257	1.657	0.441	2.416	1.974
	0.235	0.726	0.491	0.188	0.774	0.585	0.261	0.820	0.559	0.208	0.874	0.666
200	0.524	1.879	1.355	0.394	2.008	1.614	0.544	1.906	1.362	0.414	2.037	1.623
	0.835	1.908	1.073	0.732	2.011	1.279	0.953	2.065	1.112	0.847	2.172	1.325
	0.346	0.692	0.346	0.312	0.725	0.413	0.333	0.666	0.333	0.301	0.698	0.397
300	0.626	1.733	1.108	0.520	1.839	1.320	0.663	1.784	1.122	0.555	1.892	1.337
	0.986	1.912	0.926	0.897	2.001	1.104	1.059	1.975	0.916	0.971	2.063	1.092
	0.373	0.669	0.296	0.345	0.697	0.352	0.362	0.634	0.272	0.336	0.660	0.324

**Table 7:** Lower bounds (LBs), upper bounds (UBs), and average lengths (ALs) based on simple random sampling (SRS) and ranked set sampling (RSS) for Set3: (α=1.2,λ=1.5,θ=0.75).

*n*	SRS						RSS					
	90%			95%			90%			95%		
	LB	UB	AL	LB	UB	AL	LB	UB	AL	LB	UB	AL
100	0.138	2.368	2.231	−0.076	2.582	2.658	0.216	2.436	2.219	0.004	2.648	2.644
	0.607	2.218	1.611	0.453	2.372	1.919	0.740	2.385	1.645	0.583	2.542	1.960
	0.431	1.459	1.028	0.332	1.557	1.225	0.380	1.101	0.721	0.311	1.170	0.859
200	0.432	2.110	1.677	0.272	2.270	1.999	0.536	1.882	1.346	0.407	2.011	1.603
	0.936	2.159	1.223	0.819	2.276	1.457	0.946	2.056	1.110	0.840	2.162	1.322
	0.505	1.095	0.590	0.449	1.151	0.703	0.502	1.002	0.500	0.454	1.050	0.596
300	0.601	1.694	1.093	0.497	1.799	1.302	0.625	1.611	0.986	0.531	1.706	1.174
	0.961	1.890	0.929	0.872	1.979	1.107	0.991	1.870	0.879	0.907	1.954	1.047
	0.560	1.014	0.454	0.516	1.057	0.541	0.570	0.989	0.419	0.530	1.030	0.500

**Table 8:** Lower bounds (LBs), upper bounds (UBs), and average lengths (ALs) based on simple random sampling (SRS) and ranked set sampling (RSS) for Set4: (α=0.5,λ=1.5,θ=0.75).

*n*	SRS						RSS					
	90%			95%			90%			95%		
	LB	UB	AL	LB	UB	AL	LB	UB	AL	LB	UB	AL
100	0.137	1.014	0.877	0.053	1.098	1.045	0.115	0.834	0.719	0.046	0.903	0.856
	0.812	2.386	1.575	0.661	2.537	1.876	0.657	2.271	1.615	0.502	2.426	1.924
	0.359	1.337	0.978	0.266	1.431	1.165	0.329	1.357	1.028	0.231	1.455	1.225
200	0.247	0.715	0.469	0.202	0.760	0.558	0.251	0.757	0.507	0.202	0.806	0.604
	1.004	2.063	1.059	0.902	2.164	1.262	0.956	2.025	1.070	0.853	2.128	1.275
	0.538	1.181	0.643	0.477	1.242	0.766	0.472	1.031	0.559	0.418	1.084	0.666
300	0.270	0.674	0.404	0.232	0.713	0.481	0.293	0.700	0.407	0.254	0.739	0.485
	1.019	1.927	0.908	0.932	2.014	1.081	1.050	1.923	0.873	0.966	2.006	1.040
	0.535	1.056	0.522	0.485	1.106	0.622	0.526	0.983	0.458	0.482	1.027	0.545

**Table 9:** Basic statistical description for the first data set.

*n*	Mean	Median	Standard Deviation	Skewness	Kurtosis
24	71.47	61.68	36.85	0.94	0.35

**Table 10:** Basic statistical description for the second data set.

*n*	Mean	Median	Standard Deviation	Skewness	Kurtosis
20	1.9	1.7	0.7	1.59	2.35

**Table 11:** Goodness-of-fit measures, maximum likelihood estimates (MLEs) and standard errors (SEs) (in parentheses) for the first data set.

Model	CVM	AD	KS	*p*-value	MLEs				
TCPW	0.0384	0.2194	0.1078	0.9429	5.1975	0.0279	1.0104	-	-
(α,λ,θ)					(1.2660)	(0.0483)	(0.3225)	-	-
KwWE	0.0717	0.3868	0.1530	0.6272	7.8198	21.5152	1.4692	0.4015	0.0051
(a,b,α,β,λ)					(3.9916)	(0.0998)	(1.0216)	(0.3623)	(0.0019)
KwW	0.0411	0.2305	0.1131	0.9145	12.8249	2.7789	0.2028	0.5722	-
(λ,c,a,b)					(2.5960)	(9.9083)	(4.1252)	(9.706634)	-
BW	0.0402	0.2282	0.1106	0.9280	11.9919	3.4218	0.1125	0.6320	-
(α,β,c,γ)					(19.5339)	(20.2379)	(0.4676)	(1.1721)	-
W	0.0615	0.3282	0.2437	0.1156	0.0021	1.4348	-	-	-
(λ,θ)					(0.0004)	(0.06016)	-	-	-

**Table 12:** Goodness-of-fit measures, maximum likelihood estimates (MLEs) and standard errors (SEs) for the second data set.

Model	CVM	AD	KS	*p*-value	MLEs				
TCPW	0.0337	0.1959	0.1086	0.9722	200.3272	3.7521	0.6613	-	-
(α,λ,θ)					(9.9442)	(1.2310)	(0.2927)	-	-
KwWE	0.0483	0.2819	0.1380	0.8408	57.5128	0.4407	34.5503	1.0974	0.0965
(a,b,α,β,λ)					(7.3437)	(0.4832)	(7.2847)	(0.5249)	(0.1460)
KwW	0.0425	0.2458	0.1274	0.9012	68.9084	0.3396	2.9571	1.3003	-
(λ,c,a,b)					(2.4681)	(0.3679)	(1.1769)	(0.6407)	-
BW	0.0407	0.2344	0.1265	0.9057	78.7504	0.3148	3.3232	1.2708	-
(α,β,c,γ)					(19.5339)	(20.2379)	(0.4676)	(1.1721)	-
W	0.1857	1.0928	0.1849	0.5007	0.1215	2.7869	-	-	-
(λ,θ)					(0.0562)	(0.4272)	-	-	-

**Table 13:** The $-\hat{\ell }$, Akaike information criterion (AIC), corrected Akaike information criterion (CAIC), Bayesian information criterion (BIC), and Hannan–Quinn information criterion (HQIC) for the first data set.

Model	$-\hat{\ell }$	AIC	CAIC	BIC	HQIC
TCPW	117.2952	240.5904	241.7904	244.1246	241.5280
KwWE	117.9379	245.8759	249.2092	251.7662	247.4386
KwW	117.3225	242.6459	244.7502	247.3572	243.8951
BW	117.3125	242.6249	244.7302	247.3371	243.8751
W	120.9310	245.8621	246.4335	248.2182	246.4872

**Table 14:** The $-\hat{\ell }$, Akaike information criterion (AIC), corrected Akaike information criterion (CAIC), Bayesian information criterion (BIC), and Hannan–Quinn information criterion (HQIC) for the second data set.

Model	$-\hat{\ell }$	AIC	CAIC	BIC	HQIC
TCPW	15.6075	37.2151	38.7151	40.2023	37.7982
KwWE	15.9309	41.8619	46.1476	46.8405	42.8337
KwW	15.7235	39.4471	42.11383	43.4300	40.2246
BW	15.6801	39.3603	42.0272	43.3432	40.1378
W	20.5864	45.1728	45.8786	47.1642	45.5615
